# Latent Toxoplasmosis in Patients With Different Malignancy: A Hospital Based Study

**DOI:** 10.4021/jocmr2010.06.375w

**Published:** 2010-06-15

**Authors:** Amal Nimir, Amizah Othman, Soon Ee, Zohdy Musa, Iffah Abd Majid, Zalikha Kamarudin, Chee Xian, Noor Hayati Isa

**Affiliations:** a Department of Pathology, Cyberjaya University College of Medical Sciences, Malaysia; b Department of Parasitology, Universiti Kebangsaan Malaysia, Malaysia

## Abstract

**Background:**

Seroprevalence of toxoplasmosis in different populations may vary according to different environments, social customs and habits. This study was designed to measure the seroprevalence of toxoplasmosis among patients with different malignancies and to ascertain the association between common risk factors and disease transmission.

**Methods:**

This Cross-sectional study was from January to April of 2009. Four Oncology wards in Hospital Universiti Kebangsaan Malaysia (HUKM) were selected as the site for undertaking the present study. The survey involved 129 patients with different malignancies. Information was gathered by using study subject information sheet and a standardized structured questionnaire. Toxoplasma was screened by a standerd ELISA commercial kit in accordance with the manufacturers instructions and performed at the Department of Microbiology, HUKM Kuala Lumpur. A result of > 51 IU/ml of anti-Toxoplasma (IgG) antibody was regarded as positive, indicating latent or pre-existing Toxoplasma infection. A result of > 51 IU/ml of anti-Toxoplasma (IgM) antibody was regarded as positive, indicating recently acquired Toxoplasma infection.

**Results:**

Total number of seropositive patients was 54 (67.6%), the mean age was 51 years (range15 - 88 years). Toxoplasma IgG positivity was highest among Malaysian (32%). Male to female ratio was almost equal. There was a statistically significant difference in seropositivity between patients living in rural areas compared to those living in urban areas, positive history of consumption of undercooked meat and/or blood transfusion (p < 0.05).

**Conclusions:**

These findings give some support to Toxoplasma screening program and health education, including promotion of a healthy lifestyle exclusively in seronegative patients in order to prevent seroconversion and the incidence of clinically evident opportunistic infection.

**Keywords:**

Toxoplasma gondii; Risk factors; Immunocompromised; seroconversion; Seroprevalence

## Introduction

Toxoplasma gondii (T. gondii) is a protozoan parasite that is endemic worldwide and is a major opportunistic pathogen in immunocompromised hosts. Infection is mainly acquired by ingestion of food, water or soil that is contaminated with oocysts shed by cats, or by eating undercooked or raw meat containing tissue cysts [1].

Primary infection is usually subclinical, but in severely immunocompromised patients it may be life-threatening. Most primary infections subsequently phase into chronic infections in which the parasite persist in tissue cysts, mainly in the brain, retina, skeletal and cardiac muscles [2]. These chronic infections probably persist indefinitely throughout the life and may remain undiagnosed until or unless it is reactivated as a result of severe immune suppression [3].

For the diagnosis of T. gondii infection, detection of the organism itself is confirmative but very difficult. Thus, most clinical laboratories use serological tests to detect antibodies against T. gondii such as the latex agglutination (LA) test, ELISA and indirect fluorescent antibody test because of its high specificity and sensitivity [4].

Seroprevalence in different populations may vary according to different environments, social customs and habits [5, 6]. Analysis of worldwide reports indicated that about 38.5% humans, 32.9% cats and 24.2% goats were seropositive for toxoplasmosis [7].

This study was designed to measure the seroprevalence of toxoplasmosis among patients with different malignancies and to ascertain the association between common risk factors and disease transmission.

## Patients and Methods

### Type of study and sampling

This was a cross-sectional study from January to April of 2009. Random method was employed to select eligible patients.

### Study site and subjects

Four Oncology wards in Hospital Universiti Kebangsaan Malaysia (HUKM) were selected as the site for undertaking the present study. The survey involved 129 patients with different malignancies.

### Demographic information

Information was gathered by using study subject information sheet and a standardized structured questionnaire. An operational definition was used for risk factors. Contact with cats is defined as the one who is owner of one or more cats, or close contact with cats by straying, playing or feeding cats. Consumption of undercooked meat is defined as the one who has the habit of eating under-cooked meat where the method of preparation could not be guaranteed. Blood transfusion was defined as one who has received blood or blood products at least 3 months prior to the study period. Institutional permission was obtained before starting our study.

### Serologic studies

Toxoplasma was screened by a standard ELISA commercial kit in accordance with the manufacturer's instructions and performed at the Microbiology Laboratory of HUKM. A result of > 51 IU/ml of anti-Toxoplasma (IgG) antibody was regarded as positive, indicating latent or pre-existing Toxoplasma infection. A result of > 51 IU/ml of anti-Toxoplasma (IgM) antibody was regarded as positive, indicating recently acquired Toxoplasma infection.

### Statistical analyses

Statistical analyses were conducted by using SPSS version 10.0 for Windows 2004. Chi-square test for significance at 95% confidence level and p value of less than 0.05 was considered statistically significant.

## Results

During the three months of the study period, 129 samples of blood were collected from different patients. One blood sample (0.77%) was positive for Toxoplasma IgM antibodies. Three patients (2.32%) were positive for both IgM and IgG, and fifty patients (38.75%) had IgG positive blood samples. Leukaemic patients contribute the highest percentage of positive IgG results (22%), followed by lymphoma (14.4%), breast cancer (7.3%), lung carcinoma (3.5%) and other malignancies (4%).

The mean age was 51 years (range15 - 88 years). The seroprevalence of toxoplasmosis was the highest in the age group of 20 - 41 years. Ethnic groups involved in this study were as follows: sixty nine Malay, fifty Chinese, four Indians and two Indonesian patients. Toxoplasma IgG positivity was highest among Malaysian (32%), followed by Chinese (17%), Indians (1%) and (0%) among the Indonesian patients. Male to female ratio was almost equal, (50.1%) male and (49.9%) female.

Table 1 shows some predisposing factors of toxoplasmosis, seropositivity rate of Toxoplasma antibody among patients living in rural areas was higher (41.3%) compared to patients living in urban areas (26.6%). There was a statistically significant difference in seropositivity between the two groups of patients (p < 0.05). Among the seropositive patients, non-laborers represent a highest percentage compared to laborers and others. However, when multivariate analysis was performed, no significant association between Toxoplasma seroprevalence and the different occupational subgroups (p > 0.05).

No statistically significant association between seropositivity and history of contact with cats (p > 0.05). On the other hand, there is a significant association between seropositivity and positive history of consumption of undercooked meat and/or blood transfusion (Table 2).

**Table 1:** Toxoplasma Seropositivity According To Residency and Occupation

Group							P value
	**Residency**						
	Urban			Rural			
Seropositive	21 (26.6%)			33 (41.3%)			0.02
Seronegative	54 (67.5)			21 (26.2%)			
	**Occupation**						
	Laborers		Non-laborers		others		
Seropositive	9 (11.3%)		29 (36.3%)		16 (20.0%)		> 0.05
Seronegative	17 (21.2%)		21 (26.3%)		37 (46.3%)		

**Table 2:** Relationship Between Toxoplasma Seropositivity and Some Risk Factors

Group	Seronegative	Seropositive	P value
History of contact with cat			
Yes	24 (30.0%)	24 (30.0%)	> 0.05
No	51 (64.3%)	30 (37.5%)	< 0.05
Consumption of undercooked meat			
Yes	21 (26.3%)	38 (47.5%)	< 0.05
No	36 (45.0%)	16 (20.0%)	< 0.05
Blood transfusion			
Yes	54 (67.5%)	25 (31.3%)	< 0.05
No	31 (38.8%)	19 (23.8%)	< 0.05

## Discussion

We found that seroprevalence of latent Toxoplasma among patients complaining of malignant diseases was 50 (40%), which indicates that primary Toxoplasma infection appears to be subclinical and prevalent throughout life. This is a high rate when compared with previous studies in Malaysia [8, 9]. Studies in different parts of the world showed varying seroprevalence, for example, 7.7% in United Kingdom [10], 13.6% in Thailand [11], 58.1% in Egypt [12] and 67% in Brazil [13]. The possible explanation for the differences could be due to the geographical variation of the precised study sites, different diagnostic methods employed in each study which have different sensitivities, and the possible risk factors contributing to the acquisition of Toxoplasma infection. It is suggested that any immunocompromised patient should request for testing of Toxoplasma infection, especially those who are at high risk to get infection or are IgM seropositive who requires close observation.

In this study, the seroprevalence of Toxoplasma IgM antibodies 4 (5.0%) were positive. Of these, only one (1.3%) suggested recent infection (only IgM antibody); an intermediate stage (IgG and IgM) was found in 3 (3.8%) patients. We followed-up each one of the patients' medical records throughout their time of hospital admission. No clinically active toxoplasmosis was identified among those patients. It may indicate that either subjects are asymptomatic, despite having a serological status indicating the recent infection, or the clinical signs and symptoms are not reliable in the diagnosis of toxoplasmosis in those patients. Therefore, we support proper guidelines for management of this silent disease to be enforced at any level of health provision, but particularly when dealing with immunocompromised patients in which this infection can be fatal.

Toxoplasmosis was considered to be acquired in early life and the prevalence increase with age and decline in later life [14]. From our study, toxoplasmosis was found to be higher in younger population compared to older age group, thus it was in agreement with the above mentioned studies.

From this study, the Malays showed higher significant association with Toxoplasma seroprevalence than their counterparts. These data suggest that the Malays are not only the major ethnic population in Malaysia but also that they are more likely to live a life style which makes them more liable to get this infection [14, 15].

Seropositivity rate of Toxoplasma antibody among patients living in rural areas was significantly higher (p < 0.05) than those who live in urban areas. Our results is in agreement with the study done in Kuala Lumpur which showed that the seropositivity of Toxoplasmosis is higher among patients staying outside the capital city than those who reside inside it [16].

The nature of occupations is known to pose a risk to infection by Toxoplasma gondii. Although non-laborers represent the highest percentage compared to laborers and other occupations, however, there was no statistical difference in seropositivity between the 3 groups of patients (p > 0.05). This can be due to the fact that non-laborers workers may not be directly involved in agricultural pursuits, but the confinement to the same environment where toxoplasmosis is being actively transmitted would have subjected them to the same risk factors [9].

The association between contact with cats and Toxoplasma seroprevalence was not found to be significantly different in previous reported studies [17, 18]. This conflicting evidence suggests either that the possibility of contact with cats might not pose a major risk for Toxoplasma infection, or a further study should be encouraged in order to identify the real association.

Interestingly, our data shows that subjects who potentially consumed undercooked meat were associated significantly with IgG seropositivity (p < 0.05). Satay is a famous dish in Malaysia, the meat is partially cooked then dipped in sweet gravy before it is served. It would be interesting if the change of eating habits toward a healthier lifestyle were adopted by those patients.

In reality, people are usually exposed to more than one risk factor with very few exceptions, therefore a multivariate analysis was performed to detect interactions as well as to estimate any independent effect of predicted single or multiple risk factors. No difference in the significant association between those who had history of blood transfusion and those who had negative history. This suggests a larger sample size is recommended to be able to determine theses association before any conclusion could be made.

In conclusion, these findings give some support to Toxoplasma screening program and health education, including promotion of a healthy lifestyle exclusively in seronegative patients in order to prevent seroconversion and the incidence of clinically evident opportunistic infection.
